# Influence of Electrospinning Setup Parameters on Properties of Polymer-Perovskite Nanofibers

**DOI:** 10.3390/polym15030731

**Published:** 2023-01-31

**Authors:** Muhammad Bkkar, Roman Olekhnovich, Arina Kremleva, Vera Sitnikova, Yakov Kovach, Nikolai Zverkov, Mayya Uspenskaya

**Affiliations:** 1 Chemical Engineering Center, ITMO University, Saint-Petersburg 197101, Russia; 2 Faculty of Control Systems and Robotics, ITMO University, Saint-Petersburg 197101, Russia; 3 Institute of Advanced Data Transfer System, ITMO University, Saint-Petersburg 197101, Russia; 4 Department of Physics, ITMO University, Saint-Petersburg 197101, Russia

**Keywords:** polymer, perovskite, nanofibers, electrospinning, setup parameters

## Abstract

Optimizing the properties of electrospun polymer-perovskite nanofibers is considered essential for improving the performance of flexible optoelectronic devices. Here, the influence of electrospinning setup parameters (i.e., electrical voltage, collector type (planar or rotary), rotation speed, as well as process time) on the properties (i.e., external structure, perovskite crystallinity, optical properties, thermal properties, the shrinkage ratio, mechanical properties, and long-term stability) of electrospun polyvinylpyrrolidone nanofibers modified with cesium lead iodide nanocrystals has been studied. The results have shown that the structure of nanofibers is related to the electrical voltage, collector rotation speed, and process duration. Perovskite crystallinity and light absorption have improved by increasing the electrical voltage or/and the process time. The polymer’s glass transition temperature is affected by the embedded perovskite and the collector’s rotation speed. The shrinkage ratio and mechanical properties of nanofibers have been controlled by the rotation speed and the electrical voltage. The shrinkage is caused by the stress created in the nanofibers during the electrospinning process. The best mechanical properties can be noticed with the rotary collector at a rotational speed of 500–750 rpm. Nanofibers have shown good long-term stability and high thermal stability. The long-term stability is inversely proportional to the value of the electrical voltage.

## 1. Introduction

Electrospun nanofibers have attracted a lot of attention in scientific and industrial communities due to their unique properties, such as light weight, flexibility, controllable properties, and low cost, which have allowed them to be widely applied in many fields, e.g., energy generation and storage, sensors, drug delivery, filtration, etc. [1,2,3,4,5,6,7,8]. The type of material (i.e., polymers and/or additives) and its properties (optical, electrical, mechanical, etc.) determine their application area [1,2,3,4,5,6,7,8]. The properties of nanofibers can be optimized by manipulating their thickness, diameter, and orientation of nanofibers (their structure) [9,10,11,12,13,14,15,16,17]. Beads and drops lead to inhomogeneous properties of nanofibers and could result in a strong drop in their properties [17,18,19]. The structure of nanofibers can be controlled by various parameters related to the electrospinning setup (e.g., an electrical voltage, a working distance, a feed rate, a needle diameter, and a collector type), the electrospinning solution, and the surrounding environment [1,2,3,4,5,6,7,8,9,10,11,12,13,14,15,16,17,18,19]. Thus, controlling electrospinning parameters is crucial for the optimal utilization of nanofibers. Recently, polymer-perovskite nanofibers have emerged as promising materials to fabricate flexible and extensible perovskite optoelectronic devices [8,20,21,22,23,24,25,26,27,28,29,30,31]. One-stage [8,20,21,22,23,24,25,26], two-stage [27,28], or core-shell [8,29,30,31] electrospinning processes have been commonly used to prepare this type of nanofibers. The one-stage electrospinning method is considered less expensive and simpler than other methods [8,20,21,22,23,24,25,26]. Metal halide perovskites, e.g., methylammonium lead halides (MAPbX_3_, X: Cl, Br, I), and cesium lead halides (CsPbX_3_), are usually used as photoactive materials [8,20,21,22,23,24,25,26,27,28,29,30,31]. All-inorganic halide perovskites are more suitable for outdoor applications, since they have higher thermal and moisture stability than organic-inorganic halide perovskites [32,33,34]. Polar solvents (e.g., DMF, DMSO, etc.) are commonly used to dissolve perovskite precursors [23,24,25,27,28,32,33,34,35]. Polyvinylpyrrolidone (PVP), polyacrylonitrile (PAN), polyvinyl alcohol (PVA), poly(methyl methacrylate) (PMMA), and other non-conductive polymers suitable for electrospinning are commonly used to facilitate the production of electrospun nanofibers and improve the environmental stability of perovskite [8,20,21,22,23,24,25,26,27,28,29,30,31,32]. PVP is a widely used polymer for fabricating these composite nanofibers due to its good solubility in polar solvents, high spinnability, thermal stability, and ability to bind perovskites, forming a stable intermediate phase or passivating the crystal surface [8,23,24,26,27,28,32,33,34,36]. The quality of this type of composite is commonly characterized by external and internal structures, optical properties, mechanical properties, thermal properties, and long-term stability [8,20,21,22,23,24,25,26,27,28,29,30,31]. Studies about the influence of electrospinning parameters on the quality of polymer-perovskite nanofibers are rare. Bohr, C. et al. [23] have studied the influence of the polymer concentration on the morphology of MAPbI_3_:PVP single-stage electrospun nanofibers and the crystallinity of perovskite. They found that an increase in the polymer concentration from 2% to 25% has led to a decrease in the perovskite crystallinity. The light absorption of the fibrillar structure was three times better than that of the nanolayer analogue. In this method, the nanocrystals are distributed in the center and near the surface of nanofibers [31,36]. In the two-stage electrospinning method [27,28], the morphology of nanofibers and perovskite crystallinity were controlled by the electrospinning parameters [27], the concentration of the PbX_2_ solution [28], and/or the immersion time in the AX solution (A: MA, Cs, etc.) [28]. High-flexible nanofibers modified with nanocrystals on the surface of nanofibers were obtained [27,28]. For electrospun core-shell MAPbI_3_:PAN nanofibers, a decrease in the thickness of the polymeric shell has led to the formation of smaller crystals in the center of nanofibers due to the fast evaporation of the solvent, increasing the crystallization rate [29]. The structural effects lead to blue-shifted photoluminescence peaks. Due to the brittle nature of perovskites, the flexibility of nanofibers increases with increasing the concentration of the polymer [8,20,21,22,23,24,25,26,27,28,29,30,31]. The incorporation of perovskite into nanofibers reduces the crystallinity and glass transition temperature (T_g_) of the polymer [31]. Despite the loss of the nanofibrillar structure, Chen L. et al. [37] discovered that increasing the electrical voltage increases the luminescence properties of CsPbX_3_: polyvinylbutyral (PVB) nanofibers annealed at 400 °C for 1 h. Inspired by the previous results, herein we study the influence of electrospinning setup parameters on the properties of PVP:CsPbI_3_ nanofibers. The latter has been fabricated by the one-stage electrospinning method. Parameters that result in a strong change in the structure of nanofibers, e.g., the electrical voltage, the collector type, and the process time, were chosen to study their influence on the outer and inner structure of nanofibers, optical properties, mechanical properties, thermal behavior, and long-term stability.

## 2. Materials and Methods

### 2.1. Materials

Polyvinylpyridine (PVP, Mw~1,300,000) and anhydrous dimethylformamide (DMF, 99.8%) were purchased from Sigma-Aldrich, Germany. Cesium iodide (CsI, 99.9%) and lead (II) iodide (PbI_2_, 99.5%) were purchased from Chemcraft, Kaliningrad, Russia. Glass substrates were purchased from Medtechnika 7, Saint-Petersburg, Russia.

### 2.2. Preparation of the Electrospinning Solution

First, a 10% PVP solution was prepared by dissolving the polymer in DMF and stirring magnetically for 5 min at a speed of 300 rpm. Perovskite precursors (i.e., CsI:PbI_2_, 1:1) were dissolved in the as-prepared polymer solution. The ratio of PVP to perovskite was 15%. The mixed solution was stirred for 60 min at 600 rpm to acquire a homogeneous solution. Solutions were made at a temperature of 27 °C and humidity of 21%. 

### 2.3. Fabrication of Electrospun Nanofibers

**Figure 1:**
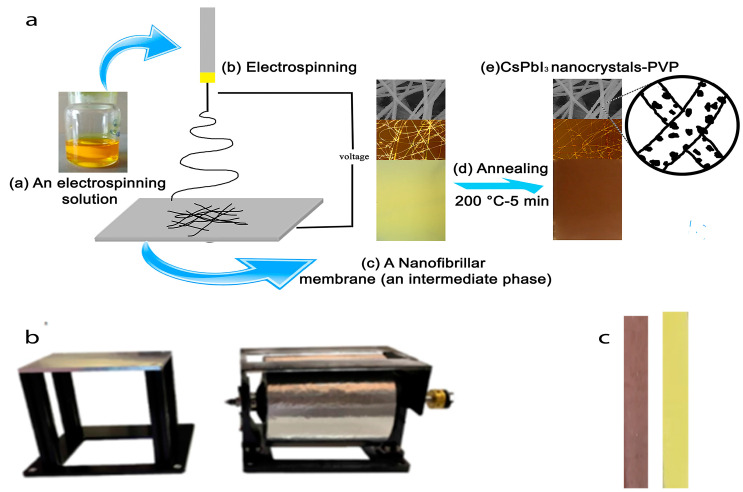
(**a**) fabrication of polymer nanofibers modified with perovskite nanocrystals; (**b**) planar and rotary collectors; (**c**) samples before and after annealing for the mechanical analysis.

A one-stage electrospinning method was used to fabricate nanofibers, which were then annealed (Figure 1a). The electrospinning solution was loaded into a 5 mL syringe at room conditions and placed into the electrospinning machine (Nanon 01A). A tubeless spinneret was used. Planar and rotary collectors were used to collect nanofibers (Figure 1b). The electrospinning process was operated at a temperature of 27 °C and a relative humidity of 21%. Samples were annealed in a laboratory oven at 200 °C for 5 min. 

### 2.4. Fabrication of Spin-Coated Layers 

The spin-coated layers were fabricated by a KW-4A spin-coater (110 V), Chemat Technology Inc., at 6000 rpm for 60 s. Glass substrates measuring 25 mm × 25 mm × 1.2 mm (L × W × H) in size were used.

### 2.5. Cleaning Substrates 

Substrates were cleaned in an ultrasonic bath UZV7/100-TH (22 kHz, 40 °C) in water, acetone, and isopropanol for 25 min. each, then dried in an oven for 2 h at 200 °C [32]. 

### 2.6. External Structure of Nanofibers

The appearance, average diameter, and orientation of nanofibers were studied using images from a scanning electron microscope (SEM) (MIRA3 TESCAN) and a microscope (Olympus STM6). The appearance of nanofibers was described using SEM images. The average diameter was calculated as the arithmetic mean of 400 nanofibers from 8 spots [32]. The average placement angle of nanofibers was calculated as the arithmetic mean of ~500 nanofibers from 3 spots. The diameter and angles of nanofibers were determined using Image J. 

### 2.7. Perovskite Crystallinity 

Perovskite crystallinity was described by X-ray diffraction (XRD) patterns, which were recorded by a DRON-8 X-ray setup in a slit configuration with a BSV-29 sharp-focus tube with a copper anode, a NaI (Tl) scintillation detector, and a β-filter (Ni).

### 2.8. Optical Properties 

The optical properties of nanofibers have been studied by recording light absorption and photoluminescence (PL) spectra in three different points of every sample. Light absorption spectra were obtained by a setup spectrometer consisting of a photonic multichannel analyzer PMA-12 (Hamamatsu) and an integrating sphere Everfine, 0.5 m, with a multi-photometer Photo-2000Z. PL spectra were recorded on an Agilent Cary Eclipse spectrofluorometer.

### 2.9. Thermal Behavior

The thermal behavior of the polymer and perovskite has been studied by thermogravimetric analysis (TGA) and differential scanning calorimetry (DSC). TGA was carried out by TG 209 F1 Libra, NETZSCH in N_2_ with a flow rate of 50 mL/min. The temperature range was 25–475 °C at a growth rate of 10 K/min. DSC was carried out by DSC 204, NETZSCH in N_2_ with a flow rate of 50 mL/min. The temperature range was 25–350 °C at a growth rate of 10 K/min. For each sample, the average results of three tests were taken.

### 2.10. Measuring the Shrinkage Ratio after Annealing 

In this case, 10 samples of 10 × 1 cm (L × W) in size were cut in both the longitudinal (LD) and transverse (TD) directions of the drum. The shrinkage ratio was calculated as ΔL/L0 (%), where ΔL the change in the length after annealing, L0 is the initial length before annealing. Next, the average value was determined.

### 2.11. Mechanical Properties

The mechanical properties were evaluated using an Instron 5943 machine. The samples were conditioned in the laboratory environment for 24 h before being tested under the same conditions. The samples were 10 cm × 1 cm (L × W) in size. The speed test was 1 mm/min. The average value was calculated for five samples in each direction.

## 3. Results

The properties of electrospun nanofibers are known to be structure dependent. Without changing the concentration of the electrospinning solution, the structure of nanofibers can be manipulated by electrospinning setup parameters (i.e., a collector type, an electrical voltage, a working distance, a needle diameter, and a feed rate) and the process time. Electrospinning setup parameters lead to a change in the average diameter of nanofibers and their orientation. The process time results in an increase in the thickness of nanofibers on the substrate. Here, the relationship between, on the one hand, the previous parameters and the structure of nanofibers, and, on the other hand, between the structure of nanofibers and its properties will be studied.

### 3.1. Electrospinning Setup Parameters and Nanofibers Structure 

**Figure 2:**
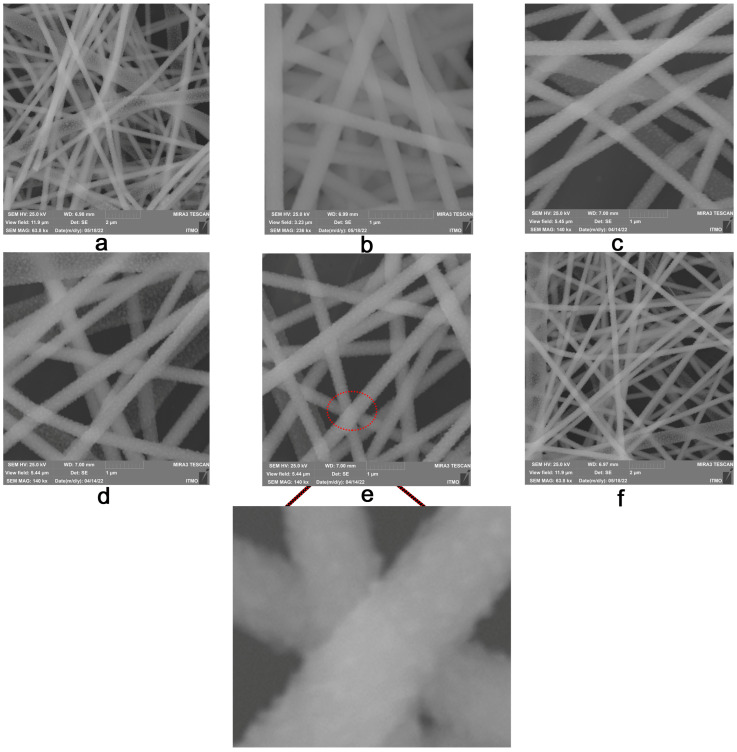
Structure of annealed nanofibers: (**a**) sample 1 and (**b**) sample 2, prepared at 20 kV and 23 kV, respectively; (**c**) sample 3 and (**d**–**f**) samples (4–6), collected using planar and rotary (250, 500, 750 rpm) collectors, respectively. Sample 1 (voltage: 20 kV, distance: 150 mm, needle diameter: 0.42 mm, feed rate: 0.1 mL/h, planar collector); Sample 2 (23 kV, 150 mm, 0.42 mm, 0.1 mL/h, planar collector); Sample 3 (20 kV, 150 mm, 0.72 mm, 0.3 mL/h, planar collector); Sample 4 (20 kV, 150 mm, 0.72 mm, 0.3 mL/h, rotary collector (250 rpm)); Sample 5 (20 kV, 150 mm, 0.72 mm, 0.3 mL/h, rotary collector (500 rpm)); Sample 6 (20 kV, 150 mm, 0.72 mm, 0.3 mL/h, rotary collector (750 rpm)).

**Figure 3:**
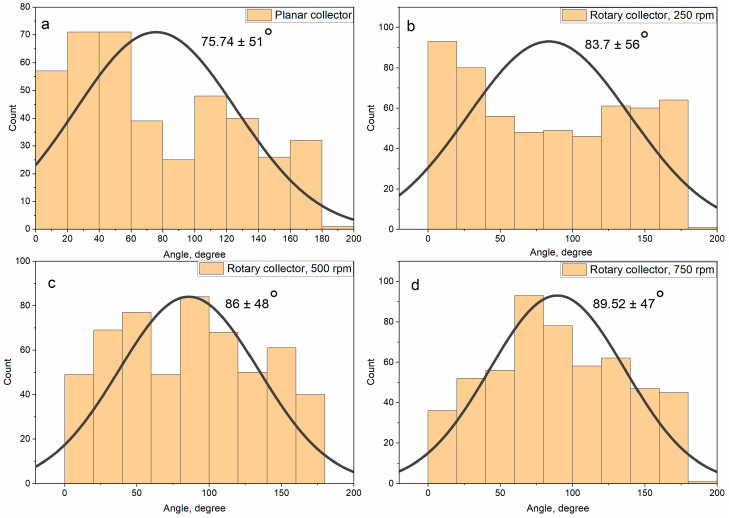
The influence of the collector type and the rotation speed on the orientation of nanofibers. Nanofibers have been collected at the following parameters: (**a**). Sample 3: 20 kV, 150 mm, 0.72 mm, 0.3 mL/h, planar collector; (**b**). Sample 4: 20 kV, 150 mm, 0.72 mm, 0.3 mL/h, rotary collector (250 rpm); (**c**). Sample 5: 20 kV, 150 mm, 0.72 mm, 0.3 mL/h, rotary collector (500 rpm); (**d**). Sample 6: 20 kV, 150 mm, 0.72 mm, 0.3 mL/h, rotary collector (750 rpm).

The structure of nanofibers can be described by their average diameter, orientation, and thickness on the substrate. In our previous work [32], a method for fabricating electrospun PVP nanofibers modified with CsPbI_3_ perovskite nanocrystals was developed (Figure 1a). The optimal electrospinning setup parameters for producing droplet-free nanofibers were identified. The applied electrical voltage was found to have a noticeable effect on the average diameter of nanofibers. Thus, this parameter was chosen to investigate its influence on the structure of nanofibers. The orientation and the thickness of nanofibers have been controlled by the collector type and the electrospinning time, respectively. The influence of electrospinning parameters on the structure of annealed nanofibers was investigated using a SEM. Table 1 presents the studied electrospinning parameters. Figure 2 shows SEM images of the resulting nanofibers. There are no noticeable changes in the external structure of nanofibers at different electrospinning parameters. Nanofibers consist of a main nanofibrillar structure that is covered with tiny fluff-like nanowires. Some split nanofibers can be seen (Figure 2a). This is owing to the high perovskite content of the nanofibers, which makes them less flexible. The average diameter drops from 329 nm to 268 nm with an increase in the electrical voltage from 20 kV to 23 kV. The effect of the collector type and rotation speed was limited to nanofibers orientation with a little change in the diameter. Figure S1 depicts micrographs and SEM images of obtained nanofibers using planar and rotary collectors (250, 500, and 750 rpm). Figure 3 shows the angular distribution and the average placement angle of nanofibers. The orientation of nanofibers increases as the rotation speed of the drum increases. In the case of nanofibers thickness, this parameter can be simply regulated by the process time. Increasing the thickness of nanofibers leads to an increase in the volume of spaces in the structure, which plays an important role in controlling its performance [8,23]. Based on the previous discussion, parameters that can lead to a noticeable change in the structure of nanofibers, such as the electrical voltage (a change in the average diameter), the collector type (a change in the orientation), and the process time (a change in the thickness), have been chosen to investigate their influence on the quality of nanofibers.

### 3.2. Electrospinning Setup Parameters and Perovskite Crystallinity

**Figure 4:**
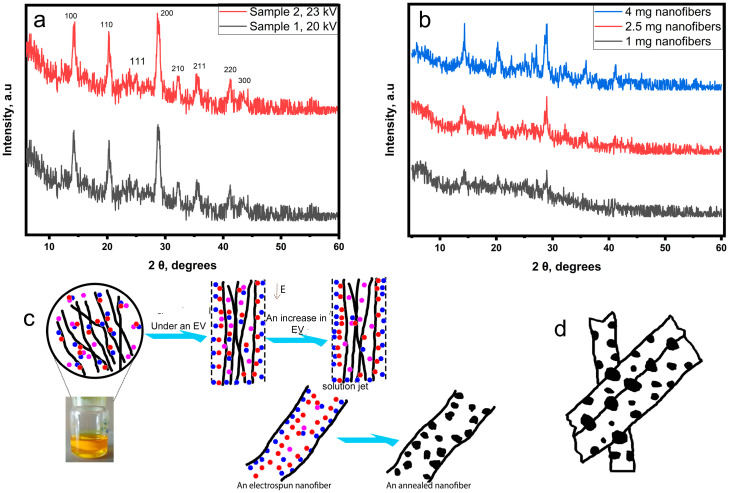
Perovskite crystallinity: (**a**) XRD patterns of samples 1 and 2, fabricated at electrical voltage values 20 kV and 23 kV, respectively (electrospinning parameters: voltage: 20–23 kV, distance: 150 mm, needle diameter: 0.42 mm, feed rate: 0.1 mL/h, planar collector); (**b**) XRD patterns of various nanofibers amounts on the glass (1, 2.5, 4 mg) (electrospinning parameters: voltage: 20 kV, distance: 150 mm, needle diameter: 0.42 mm, feed rate: 0.1 mL/h, planar collector); (**c**) a schematic illustration shows the influence of the electrical voltage on the crystallinity of perovskite; (**d**) a schematic representation of the longitudinal section of nanofibers shows the potential formation of some crystals on the combined surfaces between nanofibers.

The resulting composite nanofibers consist of a polymer-perovskite precursor complex (an intermediate phase) after electrospinning, which, after annealing, turns into polymer-perovskite nanocrystals composite [32]. XRD has been used to explore the influence of the electrospinning setup parameters on the crystallinity of perovskite. XRD results of prepared nanofibers at 20 kV and 23 kV are shown in Figure 4a. No peaks are associated with the polymer, indicating the amorphous nature of the polymer in the composite. Formed peaks around 14°, 20°, and 29° confirm the formation of the black-phase crystalline perovskite [32,33,37]. The formation of the black phase of CsPbI_3_ at a low annealing temperature is associated with the polymer that leads to the formation of a stable intermediate phase after electrospinning and lowers the energy barrier for the nucleation of perovskite [32,33,34]. The intermediate phase is formed as a result of the complexation of the polymer and the perovskite precursors. Prenucleation clusters could emerge around polymeric chains, promoting the nucleation of perovskite at low temperatures [32,35,38,39]. The intensity of peaks near 14°, 20°, and 29° increases by 2.55%, 2.78%, and 5.39%, respectively. as the electrical voltage increases from 20 kV to 23 kV, indicating an improvement in perovskite crystallinity [32]. Under the influence of the electrical field between the needle and the collector, ions can effectively migrate to the surface of the jet (Figure 4c). Ion accumulation on the surface of the solution jet stimulates the formation of perovskite nuclei near the surface that are unrestricted by polymer chains and grow larger than formed nuclei that are restricted within polymer chains [20,40,41,42,43]. Figure S2 shows the XRD results of produced nanofibers utilizing planar and rotary collectors. The results reveal that the orientation of nanofibers has no effect on the crystallinity of perovskite. In the instance of nanofibers thickness, XRD patterns of samples with various thickness are presented in Figure 4b. With increasing the thickness, perovskite crystallinity also increases. As known, an increase in the thickness of the spin-coated layer causes an increase in the crystallinity of perovskite due to the formation of larger nuclei [44]. In the case of electrospun nanofibers having a multilayer structure, the crystallinity of the perovskite also increases with their thickness. This suggests that nanofibers have reciprocal effects and that certain crystals may form on the coupled surfaces of nanofibers (Figure 4d). 

### 3.3. Electrospinning Setup Parameters and Optical Properties

**Figure 5:**
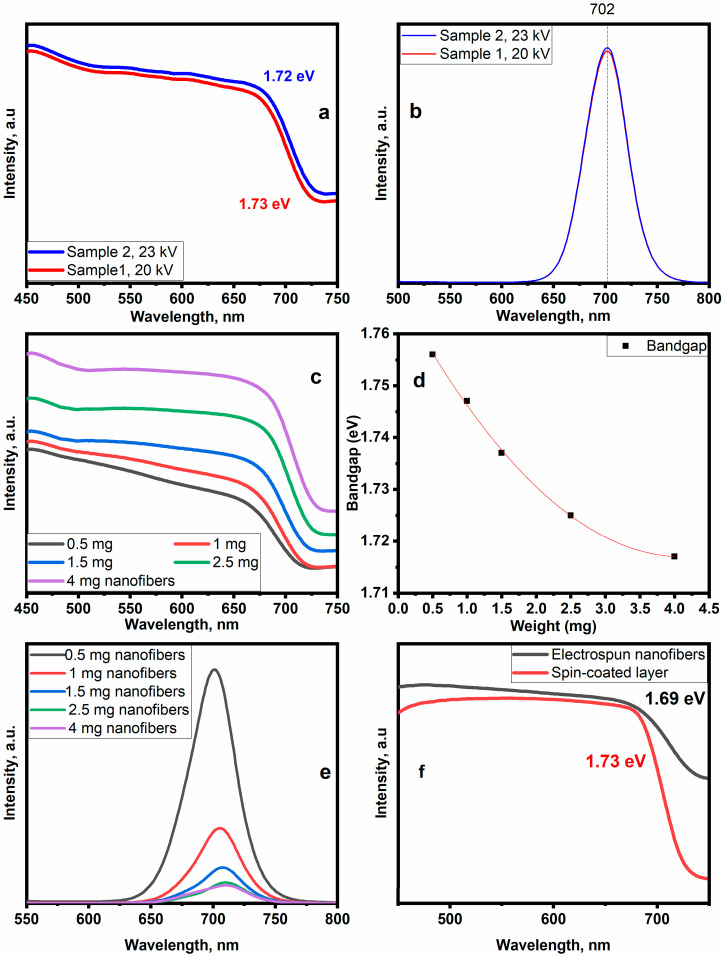
(**a**) light absorption and (**b**) PL spectra of samples 1 and 2, fabricated at 20 kV and 23 kV, respectively; (**c**) light absorption spectra of various nanofibers amounts on the glass (0.5, 1, 1.5, 2.5, 4 mg); (**d**) a bandgap-thickness relationship; (**e**) a PL-thickness relationship; (**f**) Light absorption spectra of a spin-coated layer and nanofibers for the same amount of material on the substrate. Nanofibers were fabricated at: voltage: 20 kV, distance: 150 mm, needle diameter: 0.42 mm, feed rate: 0.1 mL/h, planar collector; the spin-coated layer was fabricated at 6000 rpm for 60 s.

As the perovskite crystallinity is only affected by the electrical voltage and the thickness of nanofibers, the influence of the previous parameters on the optical properties of nanofibers has been studied by recording light absorption and PL spectra. Figure 5a,b illustrate the light absorption and PL spectra of samples 1 and 2. Figure S3 shows Tauc plots of samples 1 and 2. The light absorption spectrum almost covers the entire region of visible light. The absorption and emission intensity increases by 4.69%, 1.39%, respectively. with the increase in the electrical voltage from 20 kV to 23 kV. The optical bandgap value shifts from 1.73 eV to 1.72 eV. Regardless of the applied electrical voltage, the PL peaks remain centered at 702 nm without any changes. The improvement in light absorption and PL properties confirms XRD results on perovskite crystallinity increase. In terms of thickness, light absorption spectra are shown in Figure 5c. The results show that an increase in the thickness of nanofibers causes an increase in absorption intensity. The bandgap value reduces from 1.76 eV to 1.72 eV (Figure 5d and Figure S4), which could be attributed to the increase in grain size and optical cavities between nanofibers, leading to better light absorption properties. Red-shift PL intensity decreases with increasing the nanofibrillar layers on the substrate (Figure 5e). To assess the influence of the structure type, the light absorption spectra of the spin-coated layer and nanofibers have been taken. The results are shown in Figure 5f. Tauc plots are shown in Figure S5. The light absorption intensity of nanofibers is better due to the nanofibrillar porous structure, which results in light internal reflection and confinement [23]. This phenomenon leads to a noticeable difference in the bandgap between the two structures.

### 3.4. Electrospinning Setup Parameters and the Thermal Behavior 

**Figure 6:**
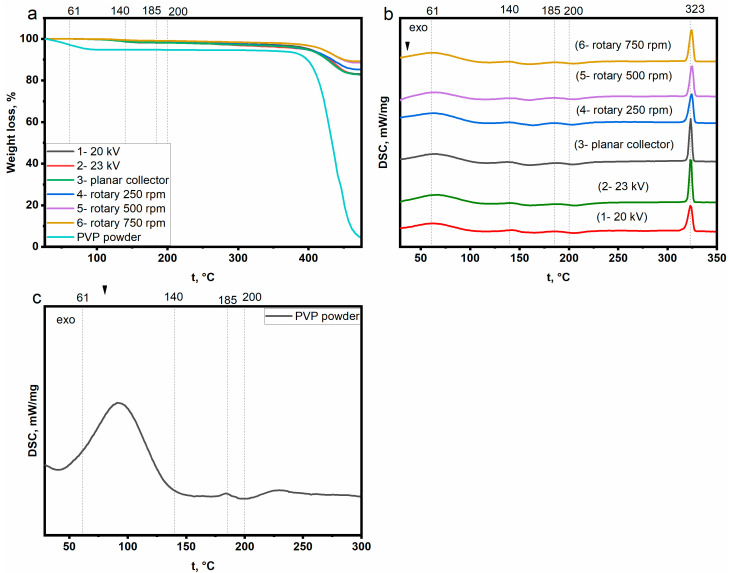
(**a**) TGA analysis; (**b**,**c**) DSC analysis. Samples were made according to the following parameters: Sample 1 (voltage: 20 kV, distance: 150 mm, needle diameter: 0.42 mm, feed rate: 0.1 mL/h, planar collector); Sample 2 (23 kV, 150 mm, 0.42 mm, 0.1 mL/h, planar collector); Sample 3 (20 kV, 150 mm, 0.72 mm, 0.3 mL/h, planar collector); Sample 4 (20 kV, 150 mm, 0.72 mm, 0.3 mL/h, rotary collector (250 rpm)); Sample 5 (20 kV, 150 mm, 0.72 mm, 0.3 mL/h, rotary collector (500 rpm)); Sample 6 (20 kV, 150 mm, 0.72 mm, 0.3 mL/h, rotary collector (750 rpm)).

The influence of electrospinning setup parameters on the thermal behavior of nanofibers has been studied by TGA and DSC. Figure 6a and Table 2 show TGA results of samples 1–6. Composite nanofibers are more stable than PVP powder. This can be explained by the role of perovskite in increasing the thermal stability of the polymer. At a temperature of 200 °C, composite nanofibers showed a weight loss of 1–2%. The weight loss (a small drop) of samples modified with perovskite at a temperature of about 140 °C is explained by the complete evaporation of the solvent residue and the onset of perovskite crystallization. 

Figure 6b and Table 3 show the DSC results of the samples (1–6). Endothermic peaks around 61 °C are attributed to dehydration. Endothermic peaks around 140 °C can indicate several processes, including the direct transition of perovskite from an amorphous to a crystalline phase, reorganization of the intermediate phase formed after electrospinning, and the full evaporation of the solvent (see the thermodynamical event around 140 °C in TGA diagrams) [31,32,33,37,38,45,46,47,48,49,50,51,52,53]. It is suggested that these processes occur sequentially. First, solvent residues complexed with perovskite precursors or PVP evaporate. Next, the reorganization of the intermediate phase and the formation of perovskite nuclei begin. This gradual change results in a wide peak in DSC diagrams and the enthalpy change at this peak represents the energy required for these processes (Table 3). Endothermic peaks around 185 °C indicate the glass transition of the polymer [54,55,56,57]. The DSC results are consistent with the XRD results regarding the amorphous nature of the polymer. The T_g_ of PVP in composite nanofibers is lower than that of the pure polymer. The distribution of perovskite into polymer nanofibers leads to an increase in the free volume not occupied by polymer chains. Thus, PVP chains can slide and move more easily at lower temperatures. In addition, a decrease in T_g_ can be noticed at a rotational speed of 500–750 rpm. This may be due to a change in the perovskite distribution into the polymer nanofibers or/and to a change in the interaction between the perovskite and the polymer, resulting in an increase in the free volume. Endothermic peaks observed around 323 °C confirm the formation of high-symmetry α-CsPbI_3_ [46,47,48,49]. Perovskite nanocrystals are formed at a temperature of 127–129 °C, and their size gradually increases with increasing temperature up to 322–324 °C [32]. It is noticed that a slight change in the crystallinity of α-CsPbI_3_ can be observed by manipulating the electrical voltage or the rotation speed of the collector. By comparing the results of XRD, light absorption, PL, and DSC, we can conclude that with small changes in the crystallinity of perovskite caused by manipulations in the structure of nanofibers, DSC may not be effective enough to accurately determine these changes. This could be attributed to various reasons:

- DSC requires a specific preparation process for the samples to be placed in the crucible, which can potentially alter the structure of the tested nanofibers and affect the accuracy of the results. In contrast, XRD, light absorption, and PL methods do not require special preparation of the samples or even handling them. They are tested directly on the substrate. Therefore, the structure of the nanofibers is well preserved. So, DSC will be more useful for detecting changes in the perovskite crystallinity when the content of polymer or perovskite is altered;
- It should also be noted that the crystallinity of perovskite can be affected by various factors such as the conditions under which it is annealed (temperature, time, atmosphere N_2_ (as in DSC) or air (usual annealing) and the annealing program (200 °C for 5 min (usual annealing) or a gradual increase in the temperature from room temperature (as in DSC technique)) [8,32,33,34,45]. These parameters can also play a role in increasing the crystallinity of perovskite in conjunction with changes in electrical voltage. Therefore, researchers in the field of perovskite-polymer composites prefer to evaluate the crystallinity of perovskite by XRD, light absorption, and PL methods, while DSC is usually used to characterize the thermal behavior of polymers.

**Table 3:** DSC results.

		PVP Powder	1–20 kV	2–23 kV	3-Planar Collector	4-Rotary 250 rpm	5-Rotary 500 rpm	6-Rotary 750 rpm
Transition, full evaporation, or/and reorganization	Temperature, °C (midpoint)	-	129.1 ± 2.8	129.4 ± 1.1	128.8 ± 3.3	129.1 ± 0.6	128.9 ± 0.3	127.1 ± 1.3
	ΔH, J/g	-	4.82 ± 0.27	4.85 ± 0.06	4.65 ± 0.21	4.7 ± 0.04	4.33 ± 0.16	3.82 ± 0.11
α-CsPbI_3_ formation	Temperature, °C (peak center)	-	322.3 ± 0.5	322.5 ± 0.3	323.5 ± 0.1	323.9 ± 0.5	323.9 ± 1.2	324.4 ± 0.2
	ΔH, J/g	-	16.42 ± 0.02	16.64 ± 0.11	16.47 ± 0.13	16.45 ± 0.13	16.47 ± 0.08	16.46 ± 0.15
	* Crystallinity, %	-	83.94 ± 0.11	85.07 ± 0.54	84.20 ± 0.65	84.10 ± 0.65	84.2 ± 0.4	84.15 ± 0.76
Polymer glass transition (T_g_)	Temperature, °C (midpoint)	179.95	174.33 ± 0.24	174.04 ± 0.14	174.53 ± 0.11	174.32 ± 0.26	173.2 ± 0.26	172.1 ± 0.97

* Crystallinity, % = ΔH_2_ × 100/ΔH_f_, ΔH_f =_ 19.56 J/g [49].

### 3.5. Electrospinning Setup Parameters and Mechanical Properties

**Figure 7:**
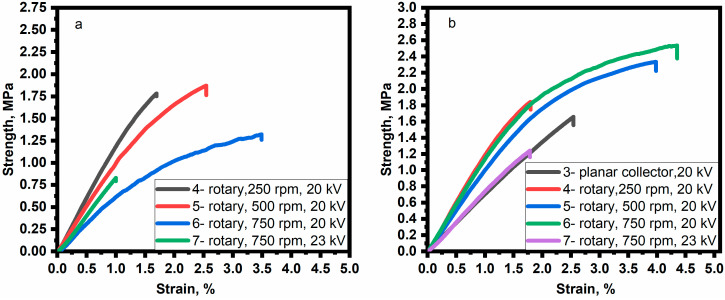
Tensile strength diagrams of specimens with a maximum tensile strength: (**a**) in the TD of the collector; (**b**) in the LD of the collector. Samples were made according to the following parameters: Sample 3 (20 kV, 150 mm, 0.72 mm, 0.3 mL/h, planar collector); Sample 4 (20 kV, 150 mm, 0.72 mm, 0.3 mL/h, rotary collector (250 rpm)); Sample 5 (20 kV, 150 mm, 0.72 mm, 0.3 mL/h, rotary collector (500 rpm)); Sample 6 (20 kV, 150 mm, 0.72 mm, 0.3 mL/h, rotary collector (750 rpm)), Sample 7 (23 kV, 150 mm, 0.72 mm, 0.3 mL/h, rotary collector (750 rpm)).

**Figure 8:**
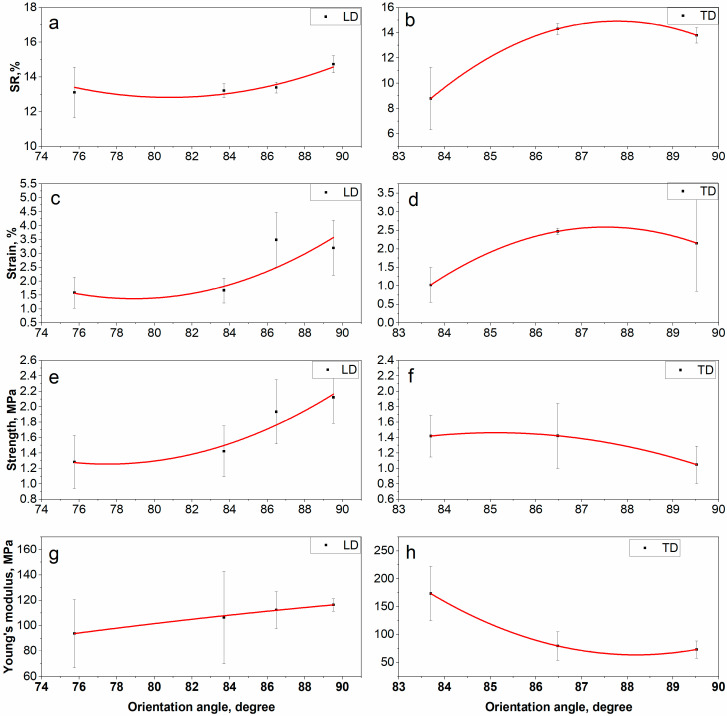
Shrinkage ratio and mechanical properties–orientation angle diagrams. (**a**) Shrinkage ratio in the LD of the collector–orientation angle; (**b**) Shrinkage ratio in the TD of the collector–orientation angle; (**c**) Strain in the LD of the collector–orientation angle; (**d**) Strain in the TD of the collector–orientation angle; (**e**) Strength in the LD of the collector–orientation angle; (**f**) Strength in the TD of the collector–orientation angle; (**g**) Young’s modulus in the LD of the collector–orientation angle; (**h**) Young’s modulus in the TD of the collector–orientation angle.

Nanofibers have been fabricated using planar and rotary collectors to study their mechanical properties. The effect of the electrical voltage on mechanical properties was studied by fabricating nanofibers with a rotary collector in order to be able to evaluate the mechanical parameters in both directions LD and TD. The sample is numbered 7 (Table 5 and Figure 7). Before studying the mechanical properties, the shrinkage ratio of the samples was determined. The results are shown in Table 4. Shrinkage ratio changes in the range of 13–15% for samples that were cut in LD and changes in the range of 9–15% for samples that were cut in TD. The shrinkage ratio in LD and TD increases as the rotation speed increases (Figure 8a,b). When the electrical voltage increases, the shrinkage ratio changes in both directions. The maximum shrinkage values are found at 500–750 rpm. This can be explained by the higher stress caused by the improved orientation and straightening of nanofibers [58,59,60]. The shrinkage is associated with the stress created in nanofibers during electrospinning. The rapid evaporation of the solvent during the electrospinning process can cause stress due to the insufficient time for polymer chains to relax. In addition, the orientation and straightening of nanofibers with a rotating collector contributes to an increase in stress. Higher stress leads to higher shrinkage after the annealing process. In addition, the annealing at temperatures above the T_g_ of the polymer results in a higher increase in shrinkage due to the increased mobility of polymer chains. Thus, a decrease in T_g_ leads to greater shrinkage [58,59,60]. All of the above explains the noticeable change in shrinkage ratio at a rotation speed of 500–750 rpm. In addition, the annealing at a temperature of 200 °C (over the T_g_ of the polymer) could lead to the formation of fiber–fiber fusions between overlapped fibers. After cooling, these fusions could form fiber–fiber bonding, increasing the mechanical properties of nanofibers [61]. 

The influence of the collector type and the electrical voltage on the mechanical properties of the resulting nanofibers was studied. The results are shown in Table 5 and Figure 7 and Figure 8. As seen, the mechanical properties of nanofibers improve with increasing the rotation speed because of the increased orientation of the nanofibers [11,14,15]. The best mechanical properties can be noticed with the rotary collector at a rotational speed of 500–750 rpm. The strain and tensile strength values in the LD are higher than those in the TD due to the difference in the orientation of nanofibers. The mechanical properties reduce as the electrical voltage increases. This may be due to an increase in the size of the crystals and a decrease in the average diameter of the nanofibers at the same time, which leads to an increase in weak points in the fiber structure, increasing the likelihood of a break. When the mechanical properties of the resulting nanofibers (strain = 1–3.5%, tensile strength = 0.7–2 MPa) are compared to those of PVP nanofibers (strain = 10–30%, tensile strength = 1–4 MPa) [62,63,64] or other PVP composite nanofibers (strain = 30–31%, tensile strength = 1.5–8 MPa) [65,66], it is clear that the resulting nanofibers have good tensile strength and low strain at break. Although the embedded perovskite prevents deformation of the samples after annealing, it contributes to a decrease in the flexibility of the nanofibers and their elongation. 

### 3.6. Long-Term Stability and an Applied Electrical Voltage

**Figure 9:**
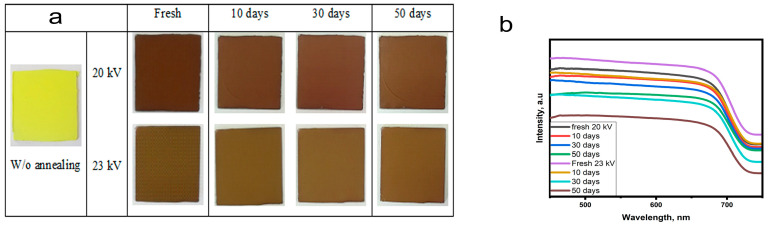
(**a**) Photos of fresh, 10-day, 30-day, and 50-day samples, fabricated at electrical voltage values 20 kV and 23 kV; (**b**) light absorption spectra of fresh, 10-day, 30-day, and 50-day samples, fabricated at electrical voltage values 20 kV and 23 kV. Samples were made according to the following parameters: voltage: 20 and 23 kV, distance: 150 mm, needle diameter: 0.42 mm, feed rate: 0.1 mL/h, planar collector).

The obtained nanofibers consist of a polymer modified with perovskite nanocrystals. The stability of black-phase perovskite at room conditions can be explained by the passivation effect of PVP and the small size of crystals [31,32,33,36,45,67]. The stability is mainly related to the average diameter of nanofibers and the distribution of nanocrystals into nanofibers. The covered crystals by the polymer will be more stable than those near the surface of nanofibers [31,32,33,36,45,67]. As the applied electrical voltage greatly affects the average diameter of nanofibers and could lead to a change in the distribution of nanocrystals into nanofibers, its influence on the stability of nanofibers has been studied. Photos of samples 1–2 after annealing are shown in Figure 9a. The color change from yellow (without annealing) to brown after annealing indicates the formation of black-phase perovskite nanocrystals. When the electrical voltage increases, the color of the samples’ changes, becoming more yellow. Based on the color change, as the electrical voltage increases, the stability of perovskite decreases. To confirm these results, changes in color and light absorption have been tracked over time in the first (an average diameter of 329 nm) and second (an average diameter of 268 nm nm) samples. Figure 9a shows photos of fresh, 10-day, 30-day, and 50-day samples. The samples appear to have undergone a little change in color over time. Figure 9b shows the light absorption spectra of fresh, 10-day, 30-day, and 50-day samples. The absorption intensity of nanofibers decreases over time, indicating the degradation of perovskite. The degradation rate of the prepared sample at 23 kV is higher than at 20 kV. Thinner nanofibers, bigger crystals, and more crystals at the surface of nanofibers result from an increase in the electrical voltage. Thus, crystals become less wrapped by the polymer, exhibiting a higher degradation rate. The electrical voltage should reach a compromise between perovskite stability and crystallinity.

## 4. Conclusions

The influence of electrospinning setup parameters on the quality of polymer-perovskite nanofibers has been studied. The increase in the electrical voltage from 20 kV to 23 kV has led to a decrease in the average diameter of nanofibers from 329 nm to 268 nm, an increase in the perovskite crystallinity and optical properties, as well as a decrease in the mechanical properties and long-term stability. The increase in crystallinity has been explained by the formation of a larger number of nuclei near the surface that are free of polymer chains and grow larger than formed nuclei that are restricted within polymer chains. The increase in process time (or the thickness of nanofibers on the substrate) has resulted in an increase in perovskite crystallinity and light absorption properties. This can be attributed to the presence of mutual effects between nanofibers and possibly the formation of some crystals on the combined surfaces between nanofibers. The bandgap value is strongly related to the porous structure between nanofibers. The thermal analysis has shown that perovskite nanocrystals are formed at a temperature of 127–129 °C, and their size gradually increases with increasing temperature up to 322–324 °C. The glass transition of the polymer happens at a temperature of 172–175 °C. Composite nanofibers showed a weight loss of 1–2% at a temperature of 200 °C. Increasing the rotation speed of the collector to 750 rpm has led to an increase in the average placement angle of nanofibers from 76° to 90°, resulting in an increase in the mechanical properties and shrinkage ratio. The main mechanism that determines the shrinkage during annealing has been revealed. The relationship between the rotation speed of the drum, the glass transition temperature of the polymer, and the shrinkage of nanofibers was described. An increase in the rotation speed in the range of 500–750 rpm led to an increase in the stress in the nanofibers and a decrease in the glass transition temperature of the polymer, resulting in a higher shrinkage ratio. However, the highest mechanical properties have been registered at a rotational speed of 500–750 rpm. Mechanical properties provide the opportunity to analyze the behavior and performance of flexible optoelectronic devices under mechanical stress. The results of this work contribute to the optimal use of electrospun nanofibers for flexible optoelectronic devices. In addition, this paper provides some illustrations that may help to better understand the relationship between the polymer and perovskite in their composites and their behavior during processing and application.

## Figures and Tables

**Table 1:** Electrospinning parameters and the average diameter of nanofibers after annealing.

Sample Number	Perovskites:Solvent, mmol	Electrospinning Parameters	Average Diameter, nm	
Sample 1	(CsI:PbI_2_:DMF)- (1:1:16.15 mmol)	Voltage: 20 kV, distance: 150 mm, Needle diameter: 0.42 mm, feed rate: 0.1 mL/h.	329 ± 70	
Sample 2		23 kV, 150 mm, 0.42 mm, 0.1 mL/h.	268 ± 48	
-		23 kV, 120 mm, 0.42 mm, 0.1 mL/h.	271 ± 55	
-		23 kV, 150 mm, 0.42 mm, 0.3 mL/h.	279 ± 51	
-		23 kV, 150 mm, 0.72 mm, 0.1 mL/h.	287 ± 92	
Sample 3		20 kV, 150 mm, 0.72 mm, 0.3 mL/h, planar collector	360.7 ± 85	
Sample 4		20 kV, 150 mm, 0.72 mm, 0.3 mL/h, rotary collector	250 rpm	358.4 ± 74
Sample 5			500 rpm	347.6 ± 70
Sample 6			750 rpm	345.5 ± 62

**Table 2:** TGA results.

	1–20 kV	2–23 kV	3-Planar Collector	4-Rotary 250 rpm	5-Rotary 500 rpm	6-Rotary 750 rpm	PVP Powder
Extrapolated onset temperature, °C	402.4	409.7	403.5	407.9	407.8	406.3	396.4
Weight loss, %	94.8	93.8	94.3	94	96.2	96.3	90.5
200 °C							
Weight loss, %	98.05	98.20	98.11	98.67	98.99	98.99	94.68

**Table 4:** The shrinkage ratio of samples 1–5.

Sample	3-planar collector, 20 kV		4-rotary 250 rpm, 20 kV		5-rotary 500 rpm, 20 kV		6-rotary 750 rpm, 20 kV		7- rotary 750 rpm, 23 kV	
Sample direction	LD	TD	LD	TD	LD	TD	LD	TD	LD	TD
Shrinkage ration, %	13.11	-	13.21	8.79	13.39	14.3	14.73	13.79	13.04	14.51
±	1.45	-	0.4	2.47	0.31	0.45	0.49	0.61	0.8	1

**Table 5:** Mechanical properties of samples 3–7.

Sample	LD/TD	Strain at Break, %	Tensile Strength, MPa	Young’s Modulus, MPa
3-planar collector, 20 kV	LD	1.59 ± 0.55	1.28 ± 0.34	93.71 ± 26.83
	TD	-	-	-
4-rotary 250 rpm, 20 kV	LD	1.67 ± 0.45	1.42 ± 0.33	106.42 ± 36.46
	TD	1.03 ± 0.47	1.42 ± 0.27	173.25 ± 48.76
5-rotary 500 rpm, 20 kV	LD	3.49 ± 0.98	1.93 ± 0.42	112.29 ± 14.59
	TD	2.47 ± 0.08	1.42 ± 0.42	79.17 ± 25.44
6-rotary 750 rpm, 20 kV	LD	3.2 ± 0.99	2.12 ± 0.33	116.30 ± 4.97
	TD	2.15 ± 1.30	1.05 ± 0.24	72.87 ± 15.39
7-rotary 750 rpm, 23 kV	LD	1.19 ± 0.43	1.04 ± 0.22	105.71 ± 19.11
	TD	0.77 ± 0.22	0.63 ± 0.22	90.44 ± 10.98

## Data Availability

Not applicable.

## Supplementary Materials

The following supporting information can be downloaded at: https://www.mdpi.com/article/10.3390/polym15030731/s1, Figure S1: Micrographs and SEM images of obtained membranes using the planar collector and the rotary collector (250, 500, and 750 rpm); Figure S2: XRD results of membranes obtained using the planar collector and the rotary collector at 250, 500, and 750 rpm; Figure S3: Tauc plots of samples 1 and 2, fabricated at 20 kV and 23 kV, respectively. Figure S4: Tauc plots of samples at a diverse thickness. Figure S5: Tauc plots of a spin-coated layer and nanofibers for the same amount of material on the substrate.
