# Supplementary material for: Influence of Electrospinning Setup Parameters on Properties of Polymer-Perovskite Nanofibers
Source: Polymers (Basel). 2023 Jan 31;15(3):731. doi: 10.3390/polym15030731 (PMC9920078; doi:10.3390/polym15030731)
Supplement: Supplementary file 1 [file polymers-15-00731-s001.zip › polymers-2148532-supplementary.pdf]

polymers

MDPI

Supplementary Materials

Influence of Electrospinning Setup Parameters on Properties of Polymer-Perovskite Nanofibers

Muhammad Bkkar [1,*], Roman Olekhnovich [2], Arina Kremleva [3], Vera Sitnikova [1], Yakov Kovach [3], Nikolai Zverkov [4] and Mayya Uspenskaya [1]

List of figures:

Figure S1. Micrographs and SEM images of obtained membranes using the planar collector and the rotary collector (250, 500, and 750 rpm). The samples have been fabricated at the following electrospinning parameters: sample 3 (20 kV, 150 mm, 0.72 mm, 0.3 ml/h, planar collector); sample 4 (20 kV, 150 mm, 0.72 mm, 0.3 ml/h, rotary

Figure S2. XRD results of membranes obtained using the planar collector and the rotary collector at 250, 500, and 750 rpm (nanofibers on the substrate = 1 mg). The samples have been fabricated at the following electrospinning parameters: sample 3 (20 kV, 150 mm, 0.72 mm, 0.3 ml/h, planar collector); sample 4 (20 kV, 150 mm, 0.72 mm, 0.3 ml/h, rotary collector (250 rpm)); sample 5 (20 kV, 150 mm, 0.72 mm, 0.3 ml/h, rotary collector (500 rpm)); sample 6 (20 kV, 150 mm, 0.72 mm, 0.3 ml/h, rotary collector (750 rpm)).

Figure S3. Tauc plots of samples 1 and 2, fabricated at 20 kV and 23 kV, respectively. The samples have been fabricated at the following electrospinning parameters: voltage: 20-23 kV, distance: 150 mm, needle diameter: 0.42 mm, feed rate: 0.1 ml/h, planar collector.

Figure S4. Tauc plots of samples at a diverse thickness. All samples have been fabricated at the following electrospinning parameters: voltage: 20 kV, distance: 150 mm, needle diameter: 0.42 mm, feed rate: 0.1 ml/h, planar collector.

Figure S5. Tauc plots of a spin-coated layer and nanofibers for the same amount of material on the substrate. Nanofibers were fabricated at the following electrospinning parameters: voltage: 20 kV, distance: 150 mm, needle diameter: 0.42 mm, feed rate: 0.1 ml/h, planar collector; the spin-coated layer was fabricated at the following parameters: 6000 rpm for 60 sec.

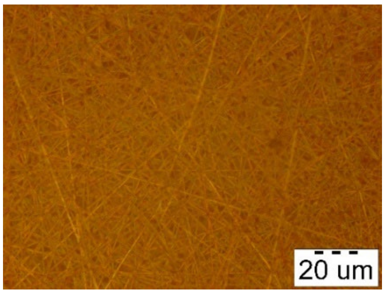

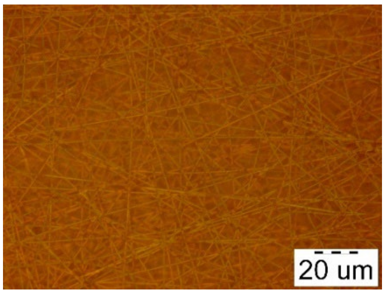

Sample 3: planar collector   Sample 4: rotary collector 250 rpm

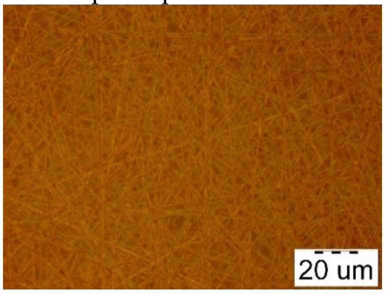

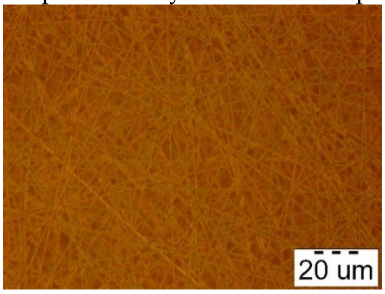

Sample 5: rotary collector 500 rpm Sample 6: rotary collector 750 rpm

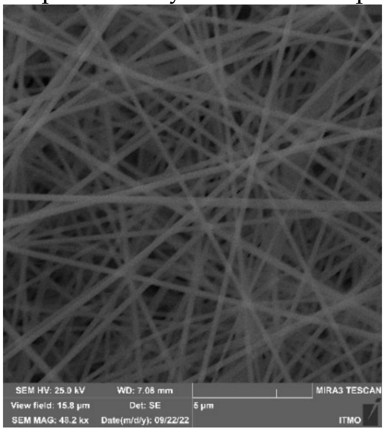

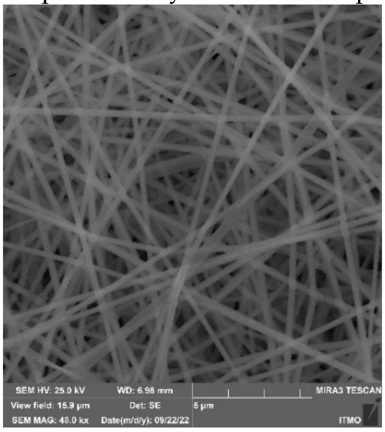

Sample 3: planar collector   Sample 4: rotary collector 250 rpm

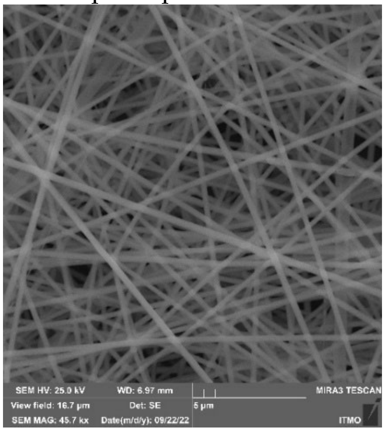

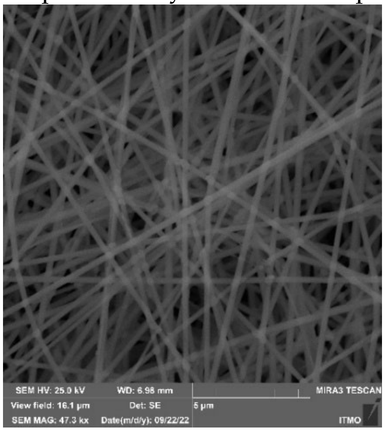

Sample 5:   rotary collector 500 rpmSample 6: rotary collector 750 rpm

Figure S1. Micrographs and SEM images of obtained membranes using the planar collector and the rotary collector (250, 500, and 750 rpm). The samples have been fabricated at the following electrospinning parameters: sample 3 (20 kV, 150 mm, 0.72 mm, 0.3 ml/h, planar collector); sample 4 (20 kV, 150 mm, 0.72 mm, 0.3 ml/h, rotary collector (250 rpm)); sample 5 (20 kV, 150 mm, 0.72 mm, 0.3 ml/h, rotary collector (500 rpm)); sample 6 (20 kV, 150 mm, 0.72 mm, 0.3 ml/h, rotary collector (750 rpm))..

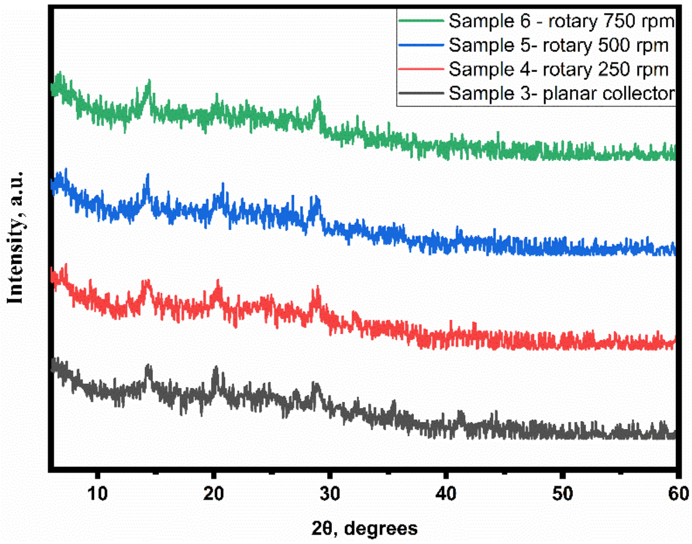

Figure S2. XRD results of membranes obtained using the planar collector and the rotary collector at 250, 500, and 750 rpm (nanofibers on the substrate = 1 mg). The samples have been fabricated at the following electrospinning parameters: sample 3 (20 kV, 150 mm, 0.72 mm, 0.3 ml/h, planar collector); sample 4 (20 kV, 150 mm, 0.72 mm, 0.3 ml/h, rotary collector (250 rpm)); sample 5 (20 kV, 150 mm, 0.72 mm, 0.3 ml/h, rotary collector (500 rpm)); sample 6 (20 kV, 150 mm, 0.72 mm, 0.3 ml/h, rotary collector (750 rpm)). .

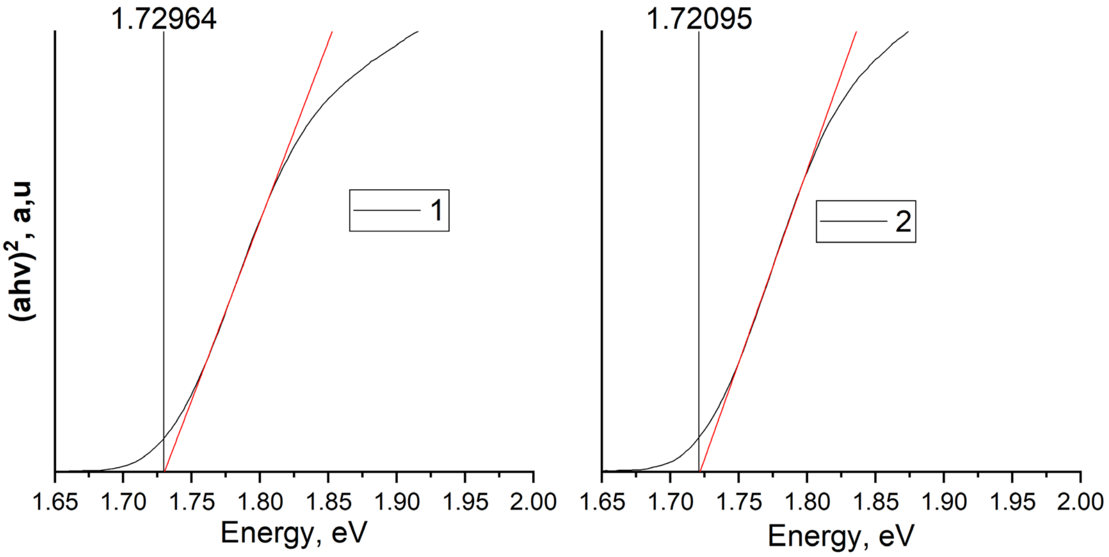

Figure S3. Tauc plots of samples 1 and 2, fabricated at 20 kV and 23 kV, respectively. The samples have been fabricated at the following electrospinning parameters: voltage: 20-23 kV, distance: 150 mm, needle diameter: 0.42 mm, feed rate: 0.1 ml/h, planar collector.

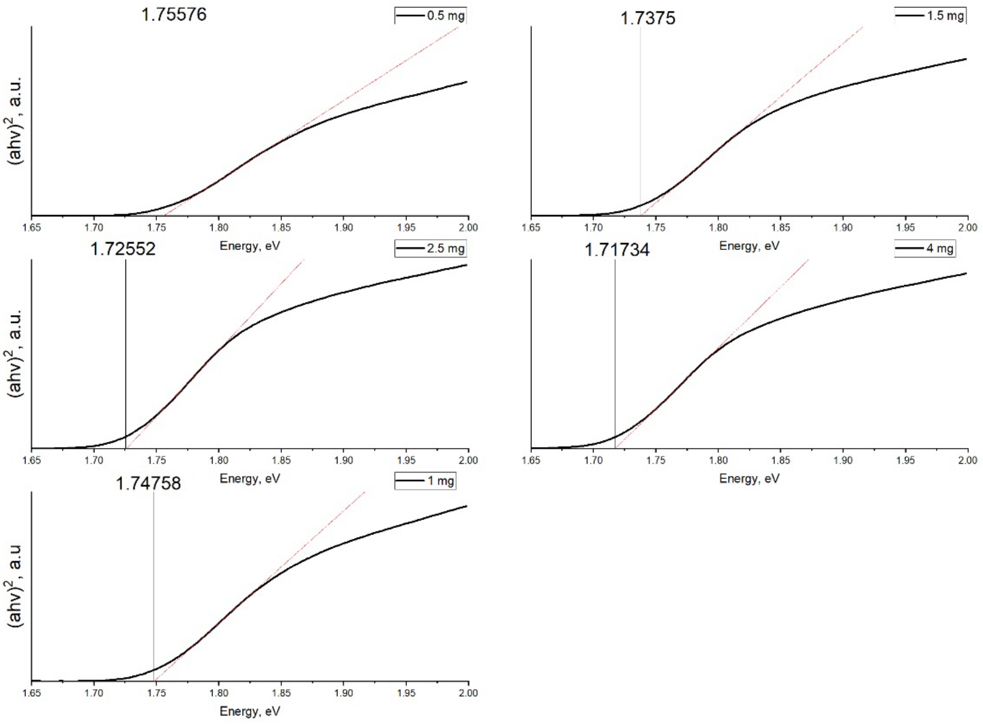

Figure S4. Tauc plots of samples at a diverse thickness. All samples have been fabricated at the following electrospinning parameters: voltage: 20 kV, distance: 150 mm, needle diameter: 0.42 mm, feed rate: 0.1 ml/h, planar collector.

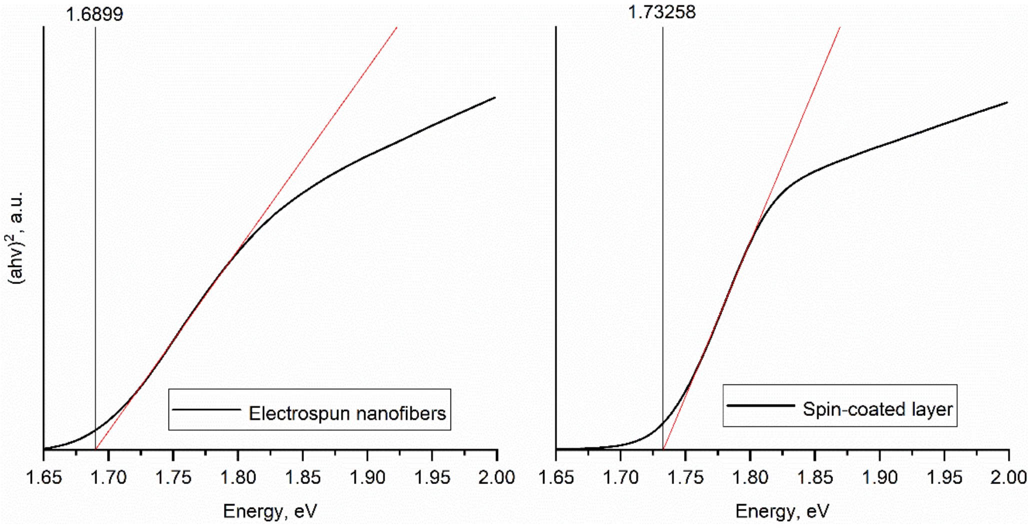

Figure S5. Tauc plots of a spin-coated layer and nanofibers for the same amount of material on the substrate. Nanofibers were fabricated at the following electrospinning parameters: voltage: 20 kV, distance: 150 mm, needle diameter: 0.42 mm, feed rate: 0.1 ml/h, planar collector; the spin-coated layer was fabricated at the following parameters: 6000 rpm for 60 sec.
